# UPLC-Q-TOF-MS/MS and Network Pharmacology Approaches to Explore the Active Compounds and Mechanisms of *Kadsura coccinea* for Treating Rheumatoid Arthritis

**DOI:** 10.3390/ijms27052097

**Published:** 2026-02-24

**Authors:** Liya Qiao, Jiashui Liao, Yongchun Huang, Ping Li, Hairong Long, Lu Chen, Tingting Tong, Xiaowen Ji, Mengli Zhang, Yude Peng, Yu Pan, Xianghua Xia

**Affiliations:** 1 Guangxi Zhuang Autonomous Region Chinese Medicinal Materials Product Quality Supervision and Inspection Station, Guangxi Botanical Garden of Medicinal Plants, Nanning 530023, China; qiaoly@gxyyzwy.com (L.Q.); liaojs@gxyyzwy.com (J.L.); huangyc@gxyyzwy.com (Y.H.); liping@gxyyzwy.com (P.L.); tongtt@gxyyzwy.com (T.T.); jixw@gxyyzwy.com (X.J.); pengyd@gxyyzwy.com (Y.P.); 2 School of Biological Science and Food Engineering, Chuzhou University, Chuzhou 239001, China; longhr83@chzu.edu.cn; 3 Guangxi Key Laboratory of High-Quality Formation and Utilization of Dao-di Herbs, National Engineering Research Center of Southwest Endangered Medicinal Resource Development, Guangxi Botanical Garden of Medicinal Plants, Nanning 530023, China; chenlu@gxyyzwy.com (L.C.); zhangml@gxyyzwy.com (M.Z.)

**Keywords:** *Kadsura coccinea*, rheumatoid arthritis, UPLC-Q-TOF-MS/MS, network pharmacology, molecular docking, computational biology

## Abstract

This study aimed to systematically identify the active constituents of *Kadsura coccinea* (Lem.) A. C. Smith (KC) and elucidate their potential mechanisms in treating rheumatoid arthritis (RA) using an integrated analytical and computational approach. Chemical profiling of KC root extract was performed by UPLC-Q-TOF-MS/MS. Active compounds and their targets were predicted using the SwissTargetPrediction database, while RA-related genes were retrieved from OMIM, GeneCards, and DisGeNET. A compound–target network was constructed and analyzed via Cytoscape. Functional enrichment analyses and protein–protein interaction (PPI) clustering were conducted to identify key pathways. Molecular docking was employed to validate interactions between core compounds and key RA targets. A total of 90 compounds were identified, primarily 36 lignans and 29 triterpenoids. Network analysis revealed 145 overlapping targets between KC and RA. These targets were further associated with 65 compounds derived from KC. Key compounds such as kadcoccinone F, kadsuralignan I and schisantherin M were linked to hub targets including MAPK14, MMPs, and JAKs, which are involved in inflammatory signaling, matrix degradation, and immune regulation. Molecular docking confirmed strong binding affinities (ΔG < −5.0 kcal/mol) between representative KC compounds and targets like MMP1, MMP2, JAK2 and JAK3, supported by analyses of hydrogen bonding, hydrophobic, and π-interactions. These results suggest that KC exerts anti-RA effects through multi-component, multi-target mechanisms, primarily modulating inflammatory signaling, immune cell recruitment, and tissue-destructive pathways. This study provides a pharmacological basis for the traditional use of KC in RA management and supports its potential as a complementary therapeutic agent.

## 1. Introduction

Rheumatoid arthritis (RA) represents a chronic, systemic autoimmune disorder characterized by progressive synovial inflammation and joint destruction, imposing a substantial global health burden. Current conventional therapies, while effective for many patients, are often associated with limited efficacy, adverse effects, and high costs, underscoring the need to explore complementary and alternative treatment strategies [1,2]. Traditional Chinese Medicine (TCM) has garnered increasing attention in this context, as it frequently employs multi-component, multi-target approaches that may synergistically modulate complex disease networks, offering potential advantages in managing multifactorial conditions such as RA [3,4,5,6].

*Kadsura coccinea* (Lem.) A.C. Smith(KC), commonly known as “Black Tiger”, is a medicinal plant whose dried roots have been historically utilized in TCM. It is traditionally described as warm, pungent, and slightly bitter, meridian-affiliated with the liver and stomach, and prescribed to promote Qi activation, invigorate blood circulation, dispel wind, and alleviate pain. In folk medicine, it has been applied in the auxiliary management of various conditions, including peptic ulcers, acute gastroenteritis, dysmenorrhea, traumatic injuries, and notably, rheumatoid arthritis [7,8]. Modern pharmacological investigations have begun to validate these traditional uses, revealing that extracts of KC possess a broad spectrum of bioactivities, including antitumor, antiviral, anti-inflammatory, anticoagulant, and hepatoprotective effects, thereby providing a scientific basis for its ethnopharmacological application [9,10,11,12,13,14]. Our previous research on its fruit polysaccharide further demonstrated significant in vitro and in vivo antioxidant and hypolipidemic activities, highlighting its potential to mitigate oxidative stress and metabolic dysregulation—processes implicated in chronic inflammatory diseases such as RA [15].

Despite documented anti-inflammatory properties and historical use in arthralgia, the specific chemical constituents of KC roots and their precise mechanisms of action in RA remain poorly characterized. In particular, systematic investigations linking KC’s chemical profile to targeted modulation of RA-associated pathways are scarce. To bridge this knowledge gap, the present study employed an integrated analytical and computational strategy. First, ultra-performance liquid chromatography coupled with quadrupole time-of-flight mass spectrometry (UPLC-Q-TOF/MS) was utilized for comprehensive metabolite profiling of the root extract. Subsequently, network pharmacology approaches were applied to construct compound-target and protein–protein interaction (PPI) networks, identifying key targets and signaling pathways implicated in RA. The functional enrichment of PPI Cluster analyses was performed to elucidate the biological processes and pathways involved. Finally, molecular docking simulations were conducted to evaluate the binding affinities and modes of interaction between the identified bioactive constituents and key RA-related targets. This multi-faceted methodology aims to decipher the pharmacochemical basis and potential mechanistic framework underlying the anti-arthritic properties of KC, providing a translational foundation for its further development as a complementary therapeutic agent for RA.

## 2. Results

### 2.1. Active Components of KC Based on UPLC-Q-TOF-MS/MS

**Figure 1:**
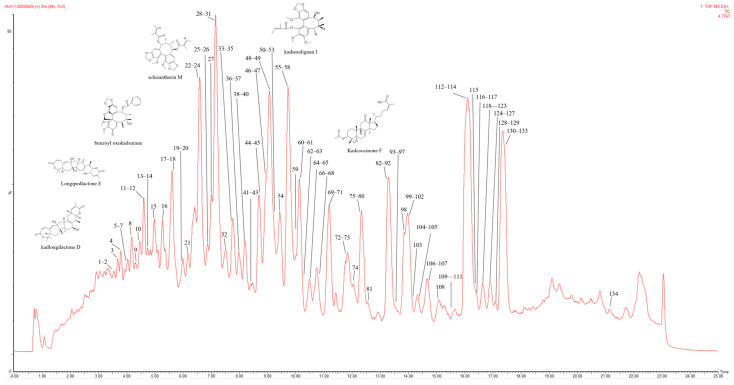
TIC of KC extract acquired in positive ionization mode by UPLC-Q-TOF-MS/MS.

Chemical profiling of the KC sample identified 90 non-redundant compounds in positive ionization mode, as shown in the total ion chromatogram (Figure 1). The compounds consisted of 36 lignans and 54 triterpenoids, with their detailed information summarized in Supplementary Table S1. The analysis revealed that lignans and triterpenoids represent the predominant chemical classes in KC. Based on the acquired mass spectrometry data, fragmentation patterns characteristic of these two compound classes were systematically characterized.

### 2.2. Structural Elucidation of Chemical Constituents in KC

Chemical profiling of *KC* indicated that lignans and triterpenoids constitute its major phytochemical classes. The structural characterization of these compounds was performed based on their acquired mass spectrometric data.

#### 2.2.1. Lignans

**Figure 2:**
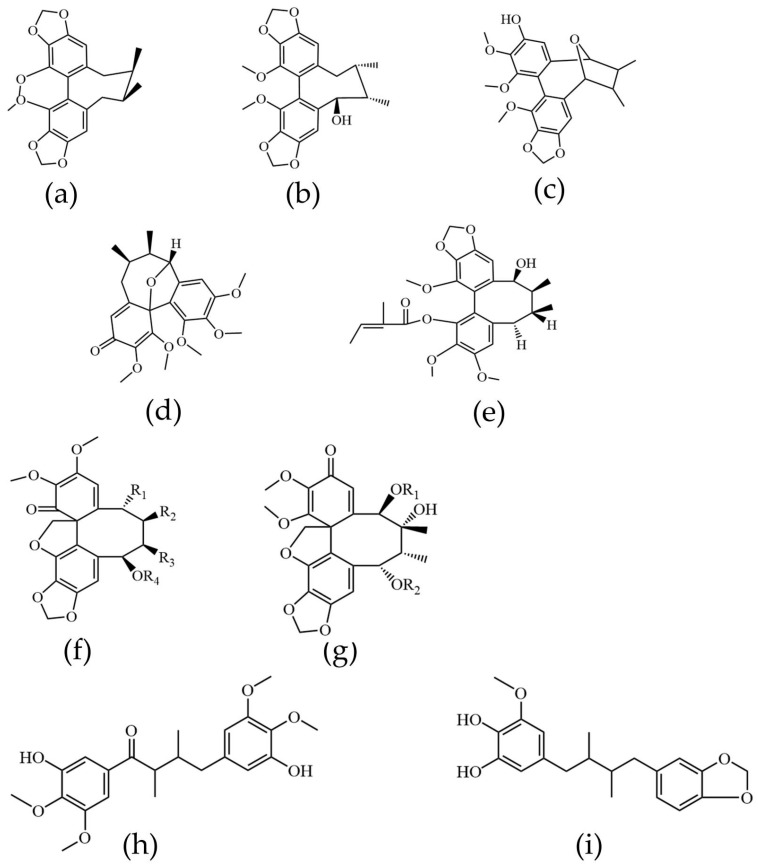
Structural elucidation of lignin-like components in KC. (**a**) R-wuweizisu, (**b**) gomisin R, (**c**) kadsulignan M, (**d**) kadsulignan A, (**e**) kadsuralignan I, (**f**) Spirobenzofuranoid dibenzocyclooctadienes lignans structure I, (**g**) Spirobenzofuranoid dibenzocyclooctadienes lignans structure II, (**h**) coccilignan A, (**i**) Kadsurindutin E.

A diverse array of lignans with unique structural skeletons was identified in KC. These compounds were classified into four primary types based on their core frameworks: dibenzocyclooctadienes, spirobenzofuranoid dibenzocyclooctadienes, diarylbutanes, and aryltetralins. Structural diversity was further enhanced by various substituents—including methoxy (-OCH_3_), methylenedioxy (-OCH_2_O-), hydroxyl (-OH), and ester (-COO-) groups—at different positions on these skeletons. Representative dibenzocyclooctadiene lignans are shown in Figure 2a–e. Compounds such as R-wuweizisu C, gomisin R, kadsulignan M, and kadsulignan A were characterized by multiple methoxy and/or hydroxyl substitutions at the C-1, C-2, and C-3 positions. In contrast, kadsuralignan I featured an angeloyl group at C-1. The core structure of spirobenzofuranoid dibenzocyclooctadiene lignans is presented in Figure 2f,g. Common features included methyl groups at C-7 and C-8. Various substituents were observed at positions C-6 and C-9, such as propyl (-Prop), acetyl (-Ac), angeloyl (-Ang), benzoyl (-Bz), and tert-butyl (-But) groups. Identified compounds of this type included schiarisanrin B, heteroclitin D, kadsulignans I, schiarisanrin A, isovaleroyl oxokadsurane, isovaleroyl oxokadsuranol, propoxyl oxokadsurane, acetoxyl oxokadsurane, and benzoyl oxokadsurane. Diarylbutane lignans (Figure 2h,i) were also detected, with examples including coccilignan A and Kadsurindutin E.

#### 2.2.2. Triterpenoids

The triterpenoid constituents of KC exhibited considerable structural diversity and complexity. Based on their carbon skeletons, they were primarily classified into three major types: lanostanes, cycloartanes, and nortriterpenoids.

**Figure 3:**
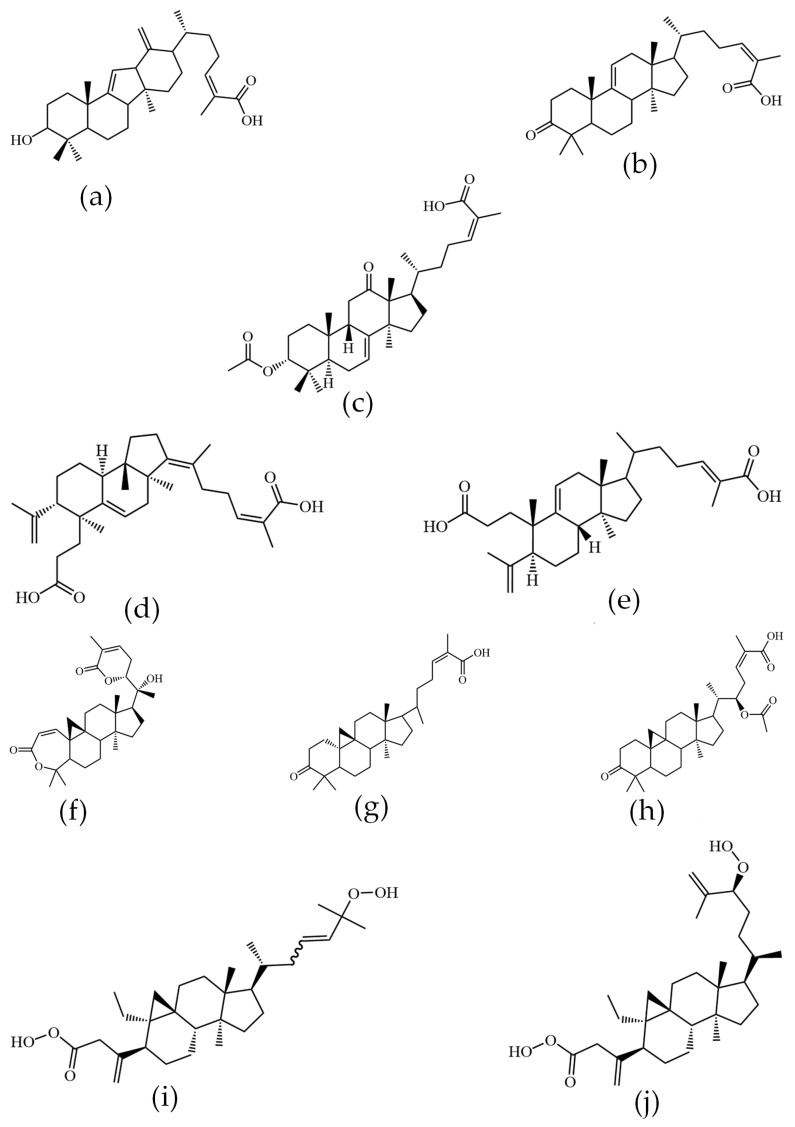
Structural elucidation of triterpenoid components in KC. (**a**) 3-hydroxy-neokadsuranic acid, (**b**) coccinic acid, (**c**) Kadcoccinone F, (**d**) Kadsuracoccinic acid A, (**e**) Kadsuracoccinic acid C, (**f**) Kadcoccilactone Q, (**g**) schisandronic acid, (**h**) heteroclic acid, (**i**) coccinetane B, (**j**) coccinetane C.

Lanostane-type triterpenoids feature a tetracyclic 24-methylcholesta-8,24(28)-diene core. Characteristic structural modifications include acetoxy (-OAc) or hydroxyl (-OH) substitutions at C-3 and C-12, double bonds located at C-9/C-11 or C-8/C-9, and a carboxyl group (-COOH) at C-26. This class is further subdivided into intact lanostanes (e.g., 3-hydroxy-neokadsuranic acid, coccinic acid, Kadcoccinone F; Figure 3a–c) and 3,4-seco-lanostanes triterpenoids (e.g., Kadsuracoccinic acid A, Kadsuracoccinic acid C; Figure 3d,e). Cycloartane-type triterpenoids share structural similarities with lanostanes but are distinguished by characteristic side-chain cleavage and skeletal rearrangements. This group includes intact cycloartanes (e.g., Kadcoccilactone Q, schisandronic acid, heteroclic acid; Figure 3f–h) and seco-cycloartanes (e.g., Coccinetane B, Coccinetane C; Figure 3i,j).

### 2.3. Active Components and Targets of KC in the Treatment of RA

**Figure 4:**
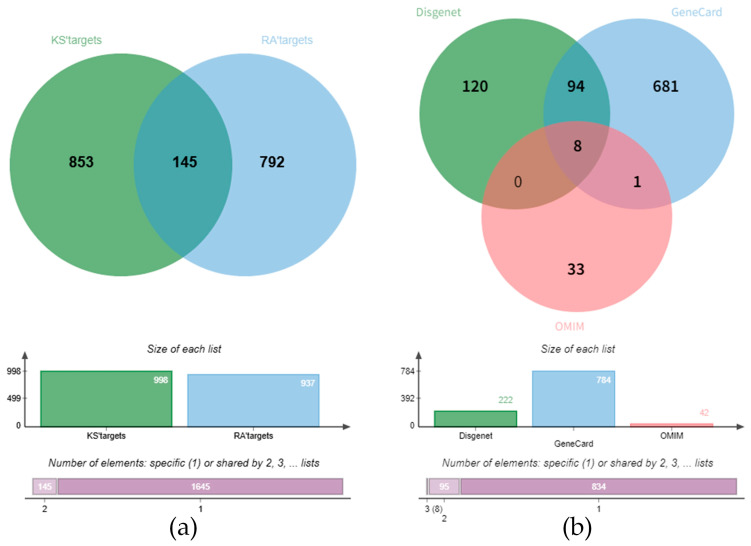
Identification of shared targets between KC and rheumatoid arthritis. (**a**) Venn diagram of overlapping targets between KC compounds and RA-related genes. (**b**) Intersection of RA-associated genes obtained from DisGeNET, GeneCards, and OMIM databases.

A total of 948 RA-related genes were compiled from public databases, including 784 from OMIM, 42 from GeneCards, and 222 from Disgenet. Concurrently, 90 compounds from KC were systematically identified by liquid chromatography–mass spectrometry, corresponding to 937 non-redundant target genes. Bioinformatics-based intersection of these gene sets revealed 145 overlapping targets, representing putative common loci of action between KC and RA (Figure 4a,b). From this pool, 65 key compounds with potential therapeutic relevance were shortlisted, comprising 36 lignans and 29 triterpenoids, which may represent promising candidates for further investigation in RA therapy (Supplementary Table S2).

### 2.4. PPI Network Cluster Analysis

**Figure 5:**
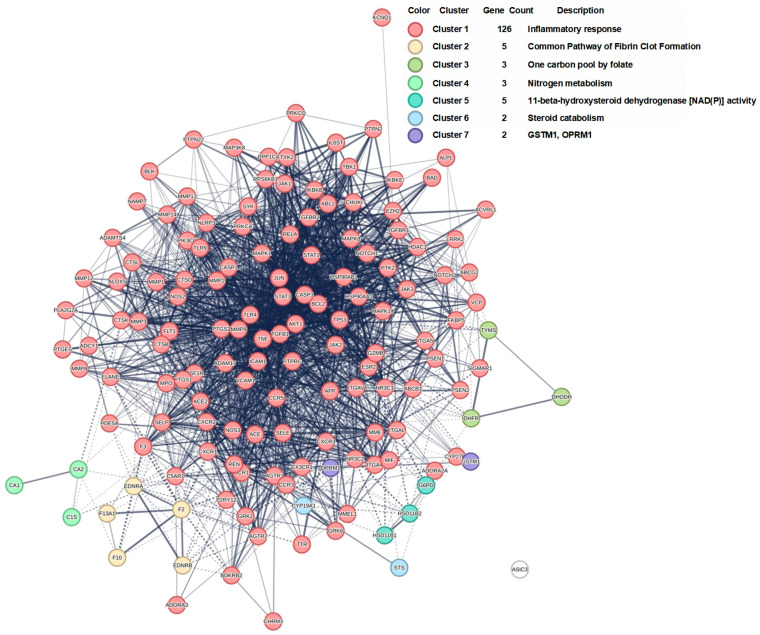
PPI network analysis. MCODE analysis revealing distinct clusters among the 145 common target proteins. Dashed lines indicate weaker protein–protein interactions, while solid lines represent stronger interactions. Increased line overlap and darker colors reflect closer connections and stronger associations among proteins.

**Figure 6:**
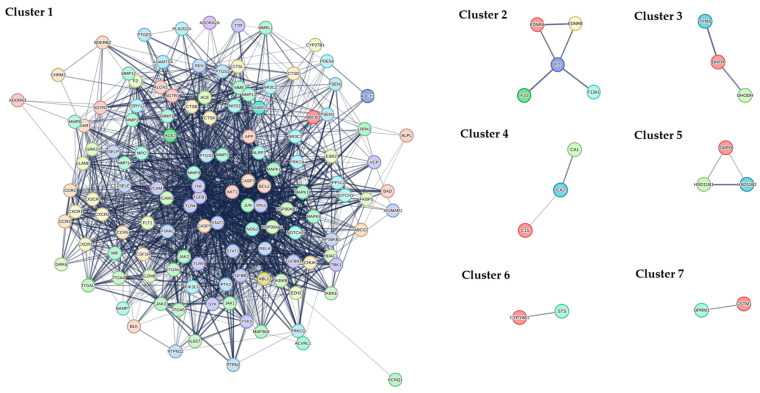
Seven functional clusters of PPI network. Solid lines represent stronger interactions between proteins from different clusters. Increased line overlap, thicker lines, or darker colors indicate closer connections and stronger associations among proteins across clusters.

The PPI network constructed from the 145 shared targets was analyzed using the MCL, which resolved it into seven distinct functional modules (Figure 5 and Figure 6). A pathologically oriented annotation reveals their specific roles in RA pathogenesis. Cluster 1, dominated by inflammatory and immune signaling hubs (e.g., MAPK14, RELA, JAK-STAT genes), encapsulates the core synovial inflammatory cascade driving leukocyte infiltration, cytokine production, and fibroblast-like synoviocyte (FLS) activation, thereby directly promoting synovitis and pannus formation. Cluster 2, enriched for coagulation factors (F2, F10) and endothelin receptors, underscores the interplay between inflammation and thrombosis in RA synovial microvasculature, contributing to ischemic injury and perpetuating a pro-inflammatory microenvironment. Cluster 3 links one-carbon folate metabolism (DHODH, TYMS) to cellular proliferation, potentially fueling the hyperplastic expansion of RA-FLS and immune cells. Cluster 4, involving carbonic anhydrases (CA1, CA2), may influence synovial fluid pH and tissue remodeling, affecting cartilage integrity and pain sensation. Cluster 5 centers on local glucocorticoid metabolism (HSD11B1), modulating inflammatory resolution and immune cell activity within the joint. Cluster 6 highlights steroid catabolism (CYP19A1), implicating estrogen metabolism in RA’s sexual dimorphism and immune modulation. Cluster 7, though poorly annotated, includes GSTM1 (oxidative stress) and OPRM1 (pain signaling), suggesting roles in redox-mediated tissue damage and nociception.

### 2.5. Integrated Enrichments Analysis of PPI Cluster 1 Reveals a Coordinated Pathogenic Network in RA

**Figure 7:**
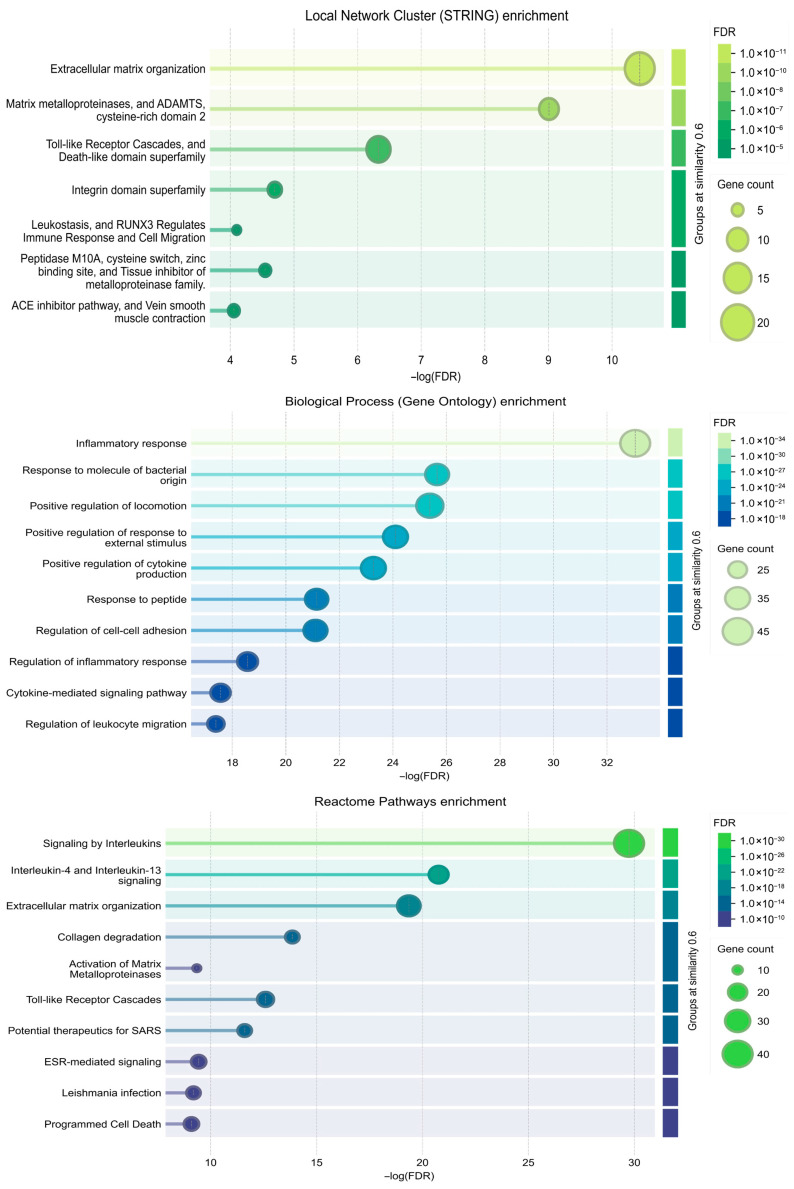
Functional enrichment and pathways analysis of PPI Cluster 1.

To elucidate the biological functions of the key module identified from the PPI network, comprehensive enrichment analysis was performed on Cluster 1, which comprised 126 genes. The results delineated a highly coordinated pathogenic network directly relevant to the core pathological processes of RA (Figure 7). Cluster 1 was significantly enriched in biological processes and molecular functions central to RA pathogenesis. Local Network Cluster Enrichment analysis identified seven significantly enriched functional groups (Figure 7). The groups “Matrix metalloproteinases, and A Disintegrin and Metalloproteinase with Thrombospondin motifs (ADAMTS), cysteine-rich domain” and “Toll-like Receptor Cascades, and Death-like domain superfamily” showed the highest statistical significance. Notably, the gene counts for all listed groups remained stable or increased across similarity thresholds from 0.6 to 1.0, indicating robust and coherent functional clustering. For instance, the “Extracellular matrix organization” group contained 13 genes, and this count was consistent across all similarity levels. The most significantly enriched biological process was “Inflammatory response”. This was followed by closely related immune and regulatory processes, including “Response to molecule of bacterial origin”, “Positive regulation of cytokine production”, “Regulation of inflammatory response”, and “Cytokine-mediated signaling pathway”. Processes concerning cell movement, such as “Positive regulation of locomotion” and “Regulation of leukocyte migration”, were also prominently enriched. Reactome Pathways Enrichment analysis placed these genes within established canonical pathways. The most significantly enriched pathway was “Signaling by Interleukins(−log(FDR) = 28.0, gene count = 40)”, highlighting a central role for cytokine signaling. Furthermore, pathways directly involved in tissue remodeling were prominent, such as “Extracellular matrix organization”, “Collagen degradation”, and “Activation of Matrix Metalloproteinases”. The enrichment of “Toll-like Receptor Cascades” corroborated the findings from the local cluster analysis.

### 2.6. Network Pharmacology Analysis Identifies Core Compounds and Target Genes in RA Treatment

**Figure 8:**
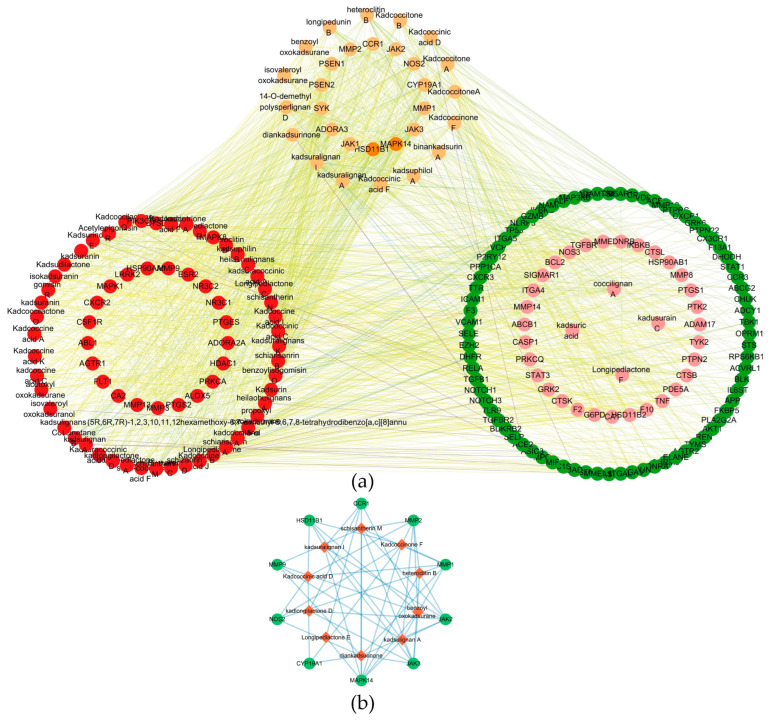
Compound–target interaction network of KC in rheumatoid arthritis. (**a**) Global network of bioactive compounds and RA-related targets. (**b**) Top 10 hub targets and their corresponding active compounds in the core network. In (**a**), target nodes are color-coded based on Degree values: orange for Degree ≥ 40, light brown for Degree ranging from 30 to 39, dark red for Degree ranging from 20 to 29, pink for Degree ranging from 10 to 19, and dark green for Degree ranging from 1 to 9, respectively.

A compound–target interaction network was constructed to systematically elucidate the therapeutic mechanisms of natural compounds in RA. Using multiple centrality metrics, the top 10 key compounds were identified as high-betweenness high-degree and nodes. These included schisantherin M, kadsuralignan I, kadsulignan A, diankadsurinone, heteroclitin B, kadcoccinone F, kadcoccinic acid D, benzoyl oxokadsurane, kadlongilactone D, and longipedlactone E. Concurrently, the top 10 hub target genes were identified: *MAPK14*, *HSD11B1*, *MMP1*, *MMP2*, *JAK3*, *CYP19A1*, *NOS2, JAK2*, *CCR1*, and *MMP9* (Table 1). These targets were functionally enriched in critical RA pathways. These pathways encompass inflammatory signaling (*MAPK14*, JAK-STAT), matrix degradation (MMPs), immune cell recruitment (*CCR1*), and metabolic regulation (*HSD11B1*, *CYP19A1*, *NOS2*). High-betweenness and High-Degree compounds, such as kadcoccinone F and kadsuralignan I, were found to interact with multiple hub target genes. Their simultaneous action on inflammatory signaling, matrix degradation, and immune cell migration explains the superior efficacy of multi-component natural medicines compared to single-target drugs. Furthermore, high neighborhood connectivity values (25–35) were observed among the hub target genes (Figure 8a,b).

### 2.7. Molecular Docking Validation

**Figure 9:**
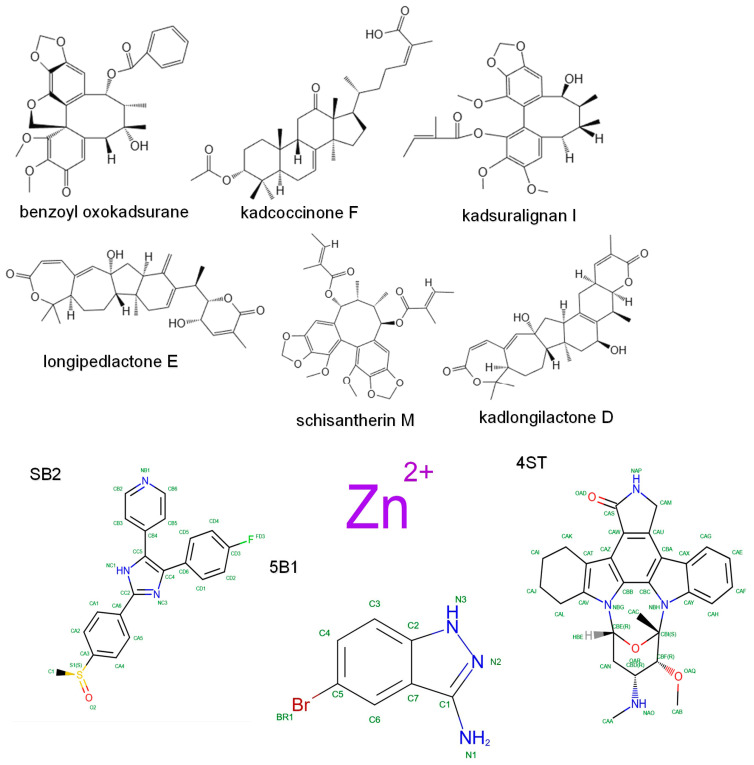
2D structures of six potential active small molecules of KC and five co-crystallized ligands of the positive control drug.

**Figure 10:**
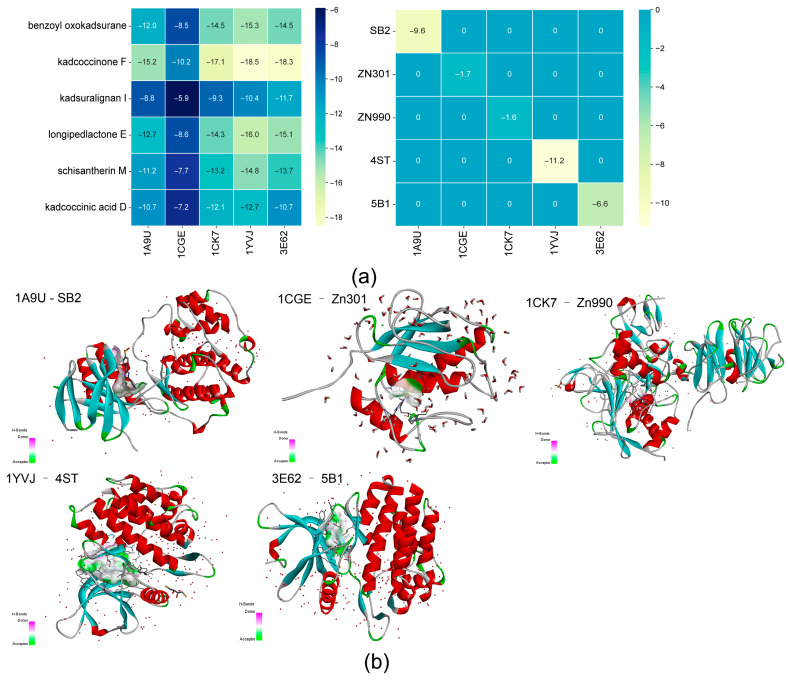
Molecular docking validation of key compound–target pairs. (**a**) Heatmap of binding energies (kcal/mol) obtained from molecular docking simulations. (**b**) Molecular docking of five co-crystallized ligands with the RA-associated target proteins. Notes: 1A9U: MAPK14; 1CGE: MMP; 1CK7: MMP2; 1YVJ: JAK3; 3E62: JAK2.

Molecular docking studies were conducted to validate the key compound–target interactions prioritized by network pharmacology. Five target proteins (MAPK14, MMP1, MMP2, JAK2, and JAK3) were docked with six representative bioactive compounds from KC: longipedlactone, kadcoccinone F, benzoyl oxokadsurane, kadlongilactone D, kadsuralignan, and schisantherin M. The structures of these six KC ligands and the five co-crystallized reference ligands are listed in Table 2 and illustrated in Figure 9. The docking results demonstrated that all six KC ligands exhibited significantly stronger predicted affinity with the five target proteins than their corresponding co-crystallized reference ligands (Figure 10a). Among the reference ligands, 4ST bound to 1YVJ (JAK3) with the lowest energy (−11.2 kcal/mol). In contrast, the metal-containing reference ligands Zn301 and Zn990, bound to 1CGE (MMP1) and 1CK7 (MMP2) respectively, showed the lowest predicted affinity, well above −5 kcal/mol. The binding poses of these reference ligands are shown in Figure 10b. Notably, kadcoccinone F displayed superior binding affinity compared to the other five KC compounds across all five targets. Its strongest interactions were observed with 1YVJ (JAK3) at −18.5 kcal/mol and with 3E62 (JAK2) at −18.3 kcal/mol. In contrast, the weakest interactions among the KC compounds were observed for kadsuralignan I with 1CGE (MMP1) at −5.9 kcal/mol and with 1A9U (MAPK14) at −8.8 kcal/mol (Figure 10a).

### 2.8. Ligand–Receptor Interaction Analysis

**Figure 11:**
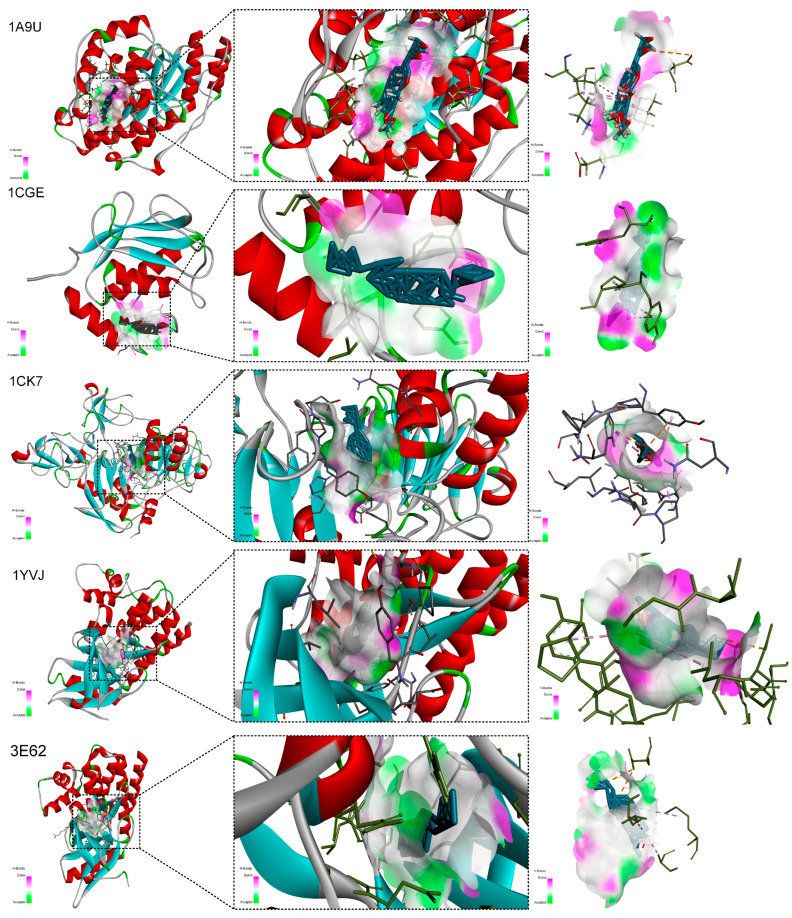
Molecular docking validation of the binding affinity between kadcoccinone F and Five RA-related target proteins. Three-dimensional views of the ligand–protein binding poses, showing both overall and close-up perspectives of the predicted binding pockets.

Analysis of the docking poses (Figure 11) indicated that kadcoccinone F bound tightly within the hydrophobic pocket of 1YVJ (JAK3). Its rigid triterpenoid scaffold formed extensive van der Waals contacts with multiple residues, including PRO105, THR56, GLY103, ASN104, ASN111, HIS193, TYR182, and PRO183. These interactions stabilized the ligand conformation within the binding site. Additional stabilization was provided by potential cation–pi and pi–sigma interactions involving aromatic or conjugated systems of the ligand and charged residues such as HIS193. Polar residues in the protein further optimized the binding orientation, contributing to the high predicted affinity.

**Figure 12:**
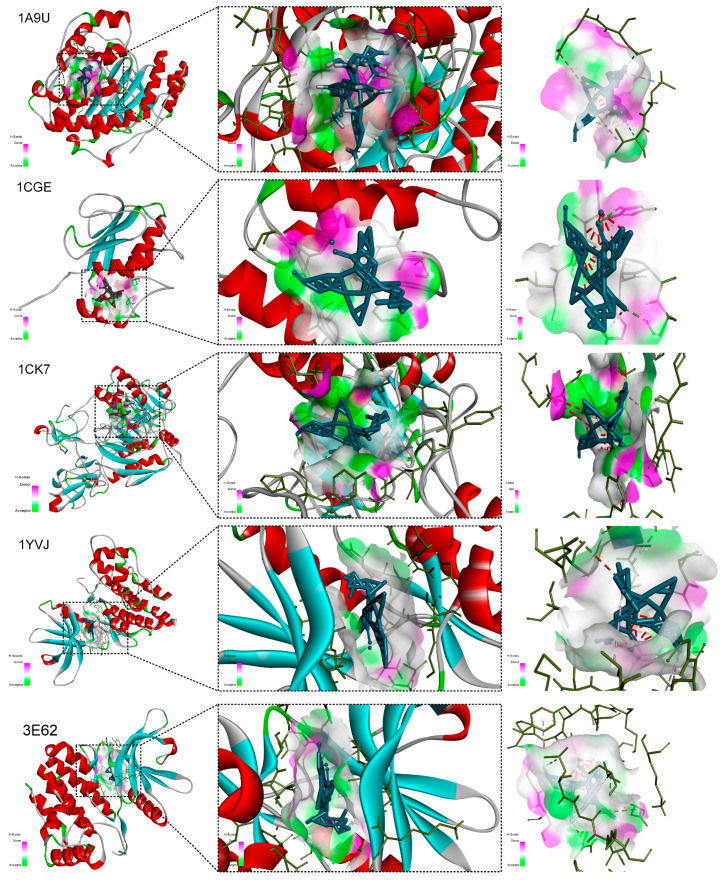
Molecular docking validation of the binding affinity between kadsuralignan I and Five RA-related target proteins. Three-dimensional views of the ligand–protein binding poses, showing both overall and close-up perspectives of the predicted binding pockets.

For kadsuralignan I, the docking poses (Figure 12) revealed efficient and specific binding to 1A9U (MAPK14). The rigid lignan skeleton was well accommodated in the hydrophobic pocket. Key interactions included hydrogen bonds formed between the ligand’s hydroxyl and methoxy oxygen atoms and polar residues (e.g., ASN, GLN, SER), which enhanced binding specificity. Extensive van der Waals contacts with surrounding hydrophobic residues (e.g., LEU, VAL, PHE) served as the primary driving force for binding. Further stability was likely provided by π-π stacking or π–cation interactions between the ligand’s aromatic rings and aromatic residues (e.g., PHE, TYR) in the active site.

## 3. Discussion

### 3.1. Integration of Analytical and Computational Strategies to Elucidate the Anti-Material Basis and Potential Mechanisms of KC

RA is a complex autoimmune disorder whose pathogenesis involves dysregulated inflammatory cascades, synovial hyperplasia, and joint destruction. While current biological and targeted synthetic disease-modifying antirheumatic drugs (DMARDs) have improved outcomes, issues of cost, accessibility, and variable patient response underscore the need for continued exploration of complementary therapeutic sources [16]. KC, traditionally used for alleviating arthralgia, presents a promising candidate. However, a systematic elucidation linking its complex chemical profile to specific anti-RA mechanisms has been lacking. This study adopted an integrative strategy combining UPLC-Q-TOF-MS/MS-based chemical profiling, network pharmacology, and molecular docking. Our findings collectively suggest that KC may exert its therapeutic effects against RA not through a single compound, but via a synergistic network of multiple bioactive lignans and triterpenoids acting on a constellation of targets across interconnected pathological pathways.

### 3.2. Structural Basis and Putative Multi-Target Engagement of KC Constituents

UPLC-Q-TOF-MS/MS analysis established a complex phytochemical profile for KC, identifying 36 lignans and 54 triterpenoids as its major constituents. The characteristic architectures of these compounds—such as the dibenzocyclooctadiene and spirobenzofuranoid dibenzocyclooctadiene skeletons in lignans and the lanostane and cycloartane frameworks in triterpenoids—are closely associated with recognized anti-inflammatory and immunomodulatory bioactivities [17]. This diverse structural repertoire forms the chemical foundation for KC’s potential polypharmacology in RA.

The innovative integration of this chemical data with network pharmacology mapped these constituents onto RA pathology. A set of 145 overlapping targets was identified and organized into seven functionally coherent protein–protein interaction modules. Topological analysis of the compound–target network prioritized several high-betweenness compounds, including kadcoccinone F, kadsuralignan I and schisantherin M. Their central network positions suggest a role as crucial hubs capable of simultaneously influencing multiple target clusters. This property may underpin a synergistic, multi-target mechanism that concurrently addresses interconnected pathological processes like inflammatory signaling and matrix degradation.

This hypothesis was further strengthened by atomistic-level validation. Molecular docking revealed that the prioritized triterpenoid kadcoccinone F had strong predicted binding affinities (ΔG < −7.0 kcal/mol) to key targets such as JAK3 and MMP2. Analysis of the binding pose with JAK3 indicated a multi-modal interaction, where its rigid scaffold formed extensive van der Waals contacts with residues like PRO105 and THR56, potentially stabilized further by a π–cation interaction with HIS193. Its high-affinity docking to MMP2, a primary executor of cartilage erosion, suggests a direct mechanism for protecting joint structure. Similarly, the lignan kadsuralignan I showed efficient predicted binding to MAPK14 driven by hydrogen bonds with polar residues (e.g., ASN, GLN) and stabilizing π-π stacking within the active site.

### 3.3. Pathophysiological Integration of PPI Network Modules in RA

The modular architecture of the PPI network provides a functional blueprint through which the multi-component extract of KC may simultaneously engage with several hallmarks of RA pathogenesis. Cluster 1, comprising 126 genes, emerges as the central effector module, densely enriched in inflammatory signaling (e.g., *MAPK14*/*p38*, JAK-STAT), immune cell recruitment (e.g., *CCR1*), and tissue-destructive pathways (e.g., MMPs). This cluster effectively encapsulates the self-amplifying loop of synovitis: from innate immune activation (e.g., via TLR cascades) and cytokine-driven signaling to the recruitment of leukocytes and the final execution of cartilage and bone degradation by matrix metalloproteinases. The network pharmacology analysis identified core compounds—notably schisantherin M, kadsuralignan I, and kadcoccinone F—as high-betweenness nodes directly linked to multiple hub targets within this cluster (e.g., MAPK14, MMP1/2/9, JAK2/3). This positions them as putative multi-target coordinators capable of concurrently dampening inflammatory signal transduction and inhibiting extracellular matrix degradation [18,19]. The subsequent molecular docking and interaction analysis lend structural credibility to this hypothesis; for instance, kadcoccinone F demonstrated strong predicted binding affinity to both JAK3 and MMP2, suggesting a potential dual mechanism of action—modulating immune cell activation via the JAK-STAT axis while directly protecting joint architecture from MMP-mediated erosion.

Clusters 2–7 suggest engagement with supporting pathological axes. Cluster 2, linked to fibrin clot formation, points to a potential modulation of the pro-thrombotic synovial microenvironment. Clusters 3 and 4, involving one-carbon folate and nitrogen metabolism, may relate to the metabolic reprogramming of proliferative synovial cells. Clusters 5 and 6, centered on glucocorticoid (HSD11B1) and estrogen (CYP19A1) metabolism, imply a possible influence on local hormonal balance affecting inflammation and its sexual dimorphism. Cluster 7, containing GSTM1 and OPRM1, hints at ancillary roles in managing oxidative stress and pain perception.

In summary, this modular analysis posits that KC may exert its effects through a functionally partitioned, multi-target strategy. The primary action is predicted via Cluster 1 to disrupt the core inflammatory-destructive cascade, while concurrent engagement with peripheral clusters could modulate the broader synovial microenvironment. This systems-level hypothesis is coherent with KC’s complex phytochemistry and is structurally supported by the strong predicted binding of its core compounds to key targets across these modules.

### 3.4. Limitations and Future Perspectives

Although mechanistic hypotheses were generated, this study remains fundamentally computational and predictive, necessitating acknowledgment of its inherent limitations. The conclusions are constrained by the potential for database bias in network pharmacology and the absence of experimental validation; furthermore, the analytical focus on organic compounds overlooks the potential synergistic role of trace elements within the botanical matrix [20]. To translate these predictions into functional evidence, a sequential validation pipeline is proposed, beginning with in vitro assays in RA-relevant models (e.g., targeting NF-κB/JAK-STAT signaling and MMP activity with prioritized compounds like kadcoccinone F), progressing to specific binding verification and subsequent in vivo assessment in arthritic models, complemented by a comprehensive phytochemical analysis that includes inorganic constituents. This rigorous, multilevel experimental strategy is essential to substantiate the therapeutic potential of KC and to advance its development as a multi-target agent for RA management.

## 4. Materials and Methods

### 4.1. Materials and Reagents

The medicinal material, KC (batch number: 121438-201503), was procured from the National Institutes for Food and Drug Control, China, and was authenticated by Professor of engineering Yude Peng from the Guangxi Botanical Garden of Medicinal Plants. The crude material was cylindrical and often curved, with a diameter ranging from 1 to 4 cm. Its surface appeared dark brown to blackish-brown and was rough in texture. The bark was frequently transversely fractured, exhibiting a beaded morphology, and could be readily separated from the wood. The texture was tough and not easily broken. The fractured surface revealed a thick bark layer containing dense, small whitish spots and fine radial striations. The wood was yellowish-white to light brown in color and displayed numerous small pores. Analytical-grade solvents including acetonitrile (MS grade, batch: 212213), formic acid (MS grade, batch: 223682), and methanol (MS grade, batch: F24OBQ203) were obtained from Thermo Fisher Scientific, Waltham, MA, USA.

### 4.2. Preparation of KC

A precisely weighed 0.3 g sample of authenticated KC crude material was placed in a sealed conical flask. Thirty milliliters of 80% ethanol solution was added, and the mixture was heated under reflux for 2 h. The 80% ethanol reflux extraction was performed in quadruplicate. To verify the consistency of the extraction, the superimposed total ion chromatogram (TIC) profiles from these four independent replicates are provided in Supplementary Figure S1. After cooling to room temperature, the extract was filtered and subsequently dried under reduced pressure. The residue was redissolved in 2 mL of methanol, and the resulting solution was filtered through a 0.22 μm microporous membrane filter prior to analysis.

### 4.3. UPLC-Q-TOF-MS/MS Experimental Instruments

The following instruments were employed in this study: an ACQUITY UPLC I-Class Plus ultra-high-performance liquid chromatography system (Waters, Milford, MA, USA); a Synapt G2-Q-TOF high-resolution mass spectrometer (Waters Co., Ltd., Milford, MA, USA); a Milli-Q Academic ultrapure water system (Merck Millipore Co., Ltd., Burlington, MA, USA); a CP225D analytical balance (Sartorius Co., Ltd., Göttingen, Germany); an HH-6 digital thermostatic water bath (Zhejiang Lichen Science Instrument Co., Ltd., Shaoxing, China); and a Hei-VAP Core ML G3 rotary evaporator (Shanghai Heidolph Co., Ltd., Shanghai, China).

### 4.4. Chromatographic Conditions

Separation was performed on a Waters ACQUITY UPLC BEH C_18_ column (100 mm × 2.1 mm, 1.7 μm) held at 40 °C. The flow rate was set at 0.3 mL/min and the injection volume was 1 μL. The mobile phase consisted of 0.1% formic acid in water (A) and acetonitrile (B). The following gradient program was applied: 0–2 min, 50–95% A; 2–20 min, 5–50% A; 20–22 min, 5% A; 22–22.1 min, 5–95% A; 22.1–25 min, 95% A.

### 4.5. Mass Spectrometric Conditions

Data were acquired in positive electrospray ionization (ESI) mode. The source parameters were as follows: drying gas flow 8 L/h, drying gas temperature 400 °C, source temperature 100 °C, cone voltage 30 V, capillary voltage 3 kV, and collision energy ramped from 20 to 40 eV. Mass spectra were recorded in MS^E^ mode over a mass range of *m*/*z* 100–1500.

### 4.6. Database Construction

A compound-specific database for KC was established by integrating information from multiple sources, including PubChem (https://pubchem.ncbi.nlm.nih.gov/) (accessed on 5 September 2025), ChemSpider (https://www.chemspider.com/) (accessed on 6 September 2025), and the Traditional Chinese Medicine Systems Pharmacology Database (TCMSP, https://www.91tcmsp.com/#/database) (accessed on 6 September 2025), supplemented by manual curation of relevant literature. The database records chemical identities, molecular formulas, structural information, relative molecular weights, and CAS registry numbers for known constituents of this botanical material.

### 4.7. Data Processing and Compound Identification

Raw UPLC-Q-TOF/MS data were processed using UNIFI software version 3.0 (Waters, Milford, MA, USA). A dedicated workflow was configured with the reference ion set at *m*/*z* 556.2771 in positive-ion mode. A mass tolerance of 8 ppm was applied, and common adduct forms (e.g., [M + H]^+^, [M + Na]^+^) were considered during spectral matching. This workflow enabled automated alignment of experimental spectra against the in-house KC’s compound library, facilitating the rapid identification of putative chemical constituents.

### 4.8. Target Collection for KC and RA

Active constituents of KC were characterized using UPLC-Q-TOF/MS analysis. Potential protein targets of these compounds were subsequently predicted using the SwissTargetPrediction database (https://www.swisstargetprediction.ch/) (accessed on 10 September 2025). In parallel, RA-associated genes were systematically retrieved from three public databases: OMIM (https://omim.org/) (accessed on 11 October 2025), GeneCards (https://genecards.org/) (accessed on 11 October 2025), and DisGeNET (https://disgenet.com/) (accessed on 11 October 2025). All acquired gene expression datasets were subjected to comprehensive preprocessing to ensure cross-platform and cross-sample comparability. Procedures included data normalization, batch effect adjustment, and filtering of low-quality probes. PPI information for RA-related proteins was obtained from the STRING (https://cn.string-db.org/, version 12.0) (accessed on 5 January 2026), BioGRID (https://thebiogrid.org) (accessed on 15 October 2025), and IntAct (https://www.ebi.ac.uk/intact/) (accessed on 15 October 2025) databases. Official gene symbols were mapped using the UniProt database (https://www.uniprot.org/) (accessed on 20 October 2025). To ensure high-quality network construction, duplicate entries, self-interactions, and low-confidence interactions were systematically excluded.

### 4.9. Functional and Pathway Enrichment Analysis

Protein–protein interaction network and functional enrichment analysis were performed using the STRING database (version 12.0). Only interactions with a combined confidence score > 0.7 were included. Functional enrichment analysis for Local Network Cluster enrichment, Gene Ontology (GO) biological processes and Reactome pathways was performed with the following thresholds: false discovery rate (FDR) < 0.05, minimum enrichment strength (log2 ratio) > 1.0. Redundant functional terms were clustered using a similarity threshold of 0.7. Functional enrichment analysis for Reactome pathways was performed on the STRING platform. Redundant pathway terms were clustered using a similarity threshold of 0.6. The results were sorted by FDR, and the top 10 most significantly enriched pathways (FDR < 0.05) were visualized.

### 4.10. Network Construction and Module Analysis

To systematically map the functional relationships among enriched terms and pathways, a network neighbor analysis was conducted using Cytoscape 3.12. This approach enabled the identification of central hub genes and pathways with potential relevance to RA pathogenesis. In parallel, a PPI network was constructed from the preprocessed interaction data, in which nodes represented proteins and edges indicated functional associations. All interactions were weighted according to confidence scores (≥0.4), interaction strength, and false discovery rate (FDR) set to Medium (5%). Network modules were subsequently identified using the Markov Cluster Algorithm (MCL), revealing functionally coherent clusters implicated in key biological processes associated with RA [21].

### 4.11. Network Construction for Bioactive Compounds and RA Targets

Shared targets between KC bioactive compounds and RA pathogenesis were identified using the Wei Sheng Xin platform (http://www.bioinformatics.com.cn) (accessed on 5 November 2025) [22]. A compound–target interaction network was subsequently constructed with Cytoscape 3.12 to visualize these relationships. Multiple network topology metrics—including Degree, Betweenness Centrality, Closeness Centrality, Stress, and Edge Betweenness—were systematically calculated. Key therapeutic targets and active compounds were then prioritized based on threshold values derived from the median and average weights of these topological indices.

### 4.12. Molecular Docking and Validation

Target proteins were prioritized based on their topological significance within the previously constructed interaction network. The two-dimensional chemical structures of key compounds were acquired from PubChem, while three-dimensional coordinates of target proteins were downloaded from the Protein Data Bank (PDB, http://www.rcsb.org) (accessed on 12 December 2025). Using MOE software (version 2022.02), water molecules and heteroatoms were removed, hydrogen atoms were added and protonated at pH 7.4, and side-chain energy minimization was performed (AMBER10:EHT forcefield, gradient cutoff 0.1 kcal/mol·Å), and correction of nonstandard residues.

Docking simulations were performed with AutoDock Vina 1.2.2. Docking grids were centered on each protein’s native co-crystallized ligand. The exhaustiveness was set to 24; other Vina parameters remained default. Detailed grid parameters are shown in Table 3. Docking accuracy was validated by re-docking each native ligand into its prepared protein using the same grid parameters. The root-mean-square deviation (RMSD) between the top docked pose and the original crystal pose was calculated in PyMOL (version 3.1). An RMSD ≤ 2.5Å was required to confirm the protocol’s reliability [23]. The resulting binding poses were visualized and analyzed using PyMOL to examine intermolecular contacts, identify key interacting residues, and illustrate ligand–protein interactions in detail. A binding energy (ΔG) below zero indicates favorable spontaneous binding. In this study, a ΔG value lower than −5.0 kcal/mol was considered to reflect significant binding activity, whereas a value below −7.0 kcal/mol was interpreted as indicative of high-affinity interaction [24].

## 5. Conclusions

In conclusion, this study provides a systematic, hypothesis-generating framework that links the complex chemical profile of KC to potential multi-target mechanisms in RA. By integrating UPLC-Q-TOF-MS/MS, network pharmacology, and computational docking, we propose that KC mediates its anti-arthritic effects through a synergistic ensemble of 36 lignans and 29 riterpenoids that collectively target inflammatory (NF-κB, MAPK, JAK-STAT) and tissue-destructive (MMP) pathways. These findings offer a modern pharmacological perspective on its traditional use and lay a predictive foundation for future experimental validation and development of KC as a complementary strategy for RA management.

## Figures and Tables

**Table 1:** Analysis of network pharmacology topological parameters of Top10 targets and core compounds of KC against RA.

Name	Degree	BC	CC	Stress	NC	Eccentricity
schisantherin M	29	0.0354	0.4142	128,948	18.2410	3
kadsuralignan I	36	0.0381	0.4259	192,554	19.7220	3
kadsulignan A	21	0.0269	0.4015	91,562	22.1900	3
diankadsurinone	31	0.0307	0.4176	140,524	21.2586	3
heteroclitin B	32	0.0311	0.4191	142,182	19.0000	3
kadcoccinone F	33	0.0292	0.4208	156,618	18.9090	3
kadcoccinic acid D	32	0.0305	0.4158	153,026	18.5000	5
benzoyl oxokadsurane	31	0.0132	0.4175	118,030	24.0000	3
kadlongilactone D	21	0.0142	0.4015	72,866	21.0000	3
longipedlactone E	24	0.0135	0.4062	86,294	21.3332	3
*MAPK14*	40	0.0344	0.4861	208,058	27.2000	4
*HSD11B1*	40	0.0353	0.4861	213,540	26.7000	4
*MMP1*	36	0.0312	0.4907	179,400	27.8611	4
*MMP2*	33	0.0247	0.4686	146,496	28.1219	4
*JAK3*	37	0.0257	0.4667	143,930	26.7297	4
*CYP19A1*	35	0.0272	0.4667	157,338	25.9429	4
*NOS2*	35	0.0252	0.4468	156,596	26.4857	4
*JAK2*	34	0.0207	0.4526	115,164	26.6176	4
*CCR1*	33	0.0216	0.4506	137,812	26.8485	4
*MMP9*	23	0.0096	0.4183	53,746	27.5652	4

BC: Betweenness Centrality. CC: Closeness Centrality. NC: Neighborhood Connectivity.

**Table 2:** Molecular docking for the five prioritized RA target proteins.

Targets	Proteins	Control Ligands PDB ID
MAPK14	Mitogen-activated protein kinase 14	SB2
MMP1	Interstitial collagenase	Zn301
MMP2	72 kDa type IV collagenase	Zn990
JAK3	Tyrosine-protein kinase JAK3	4ST
JAK2	Tyrosine-protein kinase JAK2	5B1

**Table 3:** Molecular Docking for the Five Prioritized RA Target Proteins.

Uniprot ID	PDB ID	Resolution (Å)	RMSD (Å)	Grid Size (Å) (X, Y, Z)	Center Coordinates (X, Y, Z)
Q16539	1A9U	2.50	2.113	24 × 24 × 24	2.71448, 14.6856, 28.5238
P03956	1CGE	1.90	1.986	24 × 24 × 24	−4.8946, 58.231, 57.5478
P08253	1CK7	2.80	2.352	24 × 24 × 24	44.8102, 91.6454, 145.001
O60674	3E62	1.92	1.876	24 × 24 × 24	34.0509, 40.2233, 36.3876
P52333	1YVJ	2.55	2.451	24 × 24 × 24	8.15911, −12.4665, −5.94809

## Data Availability

The data presented in this study are available on request from the corresponding author due to (specify the reason for the restriction).

## Supplementary Materials

The following supporting information can be downloaded at: https://www.mdpi.com/article/10.3390/ijms27052097/s1.

## Abbreviations

KC	*Kadsura coccinea* (Lem.) A. C. Smith.
PPI	protein–protein interaction
RA	rheumatoid arthritis
TCM	Traditional Chinese Medicine
UPLC-Q-TOF/MS	ultra-performance liquid chromatography coupled with quadrupole time-of-flight mass spectrometry
ESI	electrospray ionization
GO	Gene Ontology
FDR	false discovery rate
MCL	Markov Cluster Algorithm
PDB	Protein Data Bank
MOE	Molecular Operating Environment
MMPs	Matrixmetalloproteinases
ADAMTS	A Disintegrin and Metalloproteinase with Thrombospondin motifs
DMARDs	disease-modifying antirheumatic drugs
RMSD	root-mean-square deviation
TIC	total ion chromatogram
