# Supplementary material for: UPLC-Q-TOF-MS/MS and Network Pharmacology Approaches to Explore the Active Compounds and Mechanisms of Kadsura coccinea for Treating Rheumatoid Arthritis
Source: Int J Mol Sci. 2026 Feb 24;27(5):2097. doi: 10.3390/ijms27052097 (PMC12984762; doi:10.3390/ijms27052097)
Supplement: Supplementary file 1 [file ijms-27-02097-s001.zip › 04_Table S1.pdf]

Table S1. Chemical components identified in Kadsura coccinea (KC) by UPLC-Q-TOF-MS/MS

Number	Component name	Observed RT (min)	Formula	Neutral mass (Da)	Observed neutral mass (Da)	Adducts	Observed m/z	Mass error (ppm)	Response	Item type
1	kadlongilactone D	3.57	C30H38O6	494.26684	494.2645	+H	495.2718	-4.7	74419	triterpenoids
2	Longipedlactone E									
3	Longipedlactone A	3.87	C30H38O5	478.27192	478.2700	+H	479.2773	-3.9	136889	triterpenoids
4	Longipedlactone C	3.91	C30H40O6	496.28249	496.2811	+H, +Na	497.2884	-2.7	242914	triterpenoids
5	(5R,6R,7R)-1,2,3,10,11,12hexamethoxy-6,7-dimethyl-5,6,7,8-tetrahydrodibenzo[a,c][8]annu len-5-ol	4.04	C22H24O7	400.1522	400.1503	+H, +Na	401.1576	-4.8	209368	lignans
6	gomisin R		C22H24O7	400.1522	400.1503	+H, +Na	401.1576	-4.8	209368	lignans
7	kadsulignan M		C22H24O7	400.1522	400.1503	+H, +Na	401.1576	-4.8	209368	lignans
8	benzoyl oxokadsurane	4.18	C29H28O9	520.17333	520.1718	+H, +Na	521.1791	-2.9	95986	lignans
9	Longipedlactone F	4.33	C30H38O6	494.26684	494.266	+H	495.2732	-1.8	75220	triterpenoids
10	kadsulignan A	4.47	C23H28O7	416.1835	416.1822	+H, +Na	417.1895	-3.1	346826	lignans
11	acetoxyl oxokadsurane	4.62	C24H26O8	442.16277	442.162	+H, +Na	443.1692	-1.8	145215	lignans
12	Acetylepigomisin R		C24H26O8	442.16277	442.162	+H, +Na	443.1692	-1.8	145215	lignans

Number	Component name	Observed RT (min)	Formula	Neutral mass (Da)	Observed neutral mass (Da)	Adducts	Observed m/z	Mass error (ppm)	Response	Item type
13	KadcoccitoneA	4.75	C30H44O6	500.31379	500.3124	+H, +Na	501.3197	-2.8	170916	triterpenoids
14	kadcotrione A		C30H44O6	500.31379	500.3124	+H, +Na	501.3197	-2.8	170916	triterpenoids
15	Kadcoccilactone Q	4.76	C30H42O5	482.30322	482.3013	+H	483.3086	-4	312880	triterpenoids
16	coccilignan A	5.13	C22H28O7	404.1835	404.1846	+Na	427.1739	2.7	111670	lignans
17	isovaleroyl oxokadsurane	5.55	C27H32O9	500.20463	500.2052	+Na	523.1945	1.2	49806	lignans
18	isovaleroyl oxokadsuranol		C27H32O9	500.20463	500.2052	+Na	523.1945	1.2	49806	lignans
19	kadsulignan A	5.63	C23H28O7	416.1835	416.1822	+H, +Na	417.1895	-3.1	453879	lignans
20	Longipedlactone B	5.64	C30H40O5	480.28757	480.2867	+H, +Na	481.2939	-1.9	472988	triterpenoids
21	diankadsurinone	6.28	C23H26O7	414.16785	414.1659	+H	415.1732	-4.7	66837	lignans
22	Kadsurin	6.63	C25H30O8	458.19407	458.1937	+H	459.201	-0.8	409589	lignans
23	longipedunin B		C25H30O8	458.19407	458.1937	+H	459.201	-0.8	409589	lignans
24	schizanrin D		C25H30O8	458.19407	458.1937	+H	459.201	-0.8	409589	lignans
25	schisantherin M	6.65	C32H36O10	580.23085	580.2284	+H	581.2357	-4.2	122516	lignans
26	schisantherin N		C32H36O10	580.23085	580.2284	+H	581.2357	-4.2	122516	lignans

Number	Component name	Observed RT (min)	Formula	Neutral mass (Da)	Observed neutral mass (Da)	Adducts	Observed m/z	Mass error (ppm)	Response	Item type
27	benzoylisogomisin O	7.15	C30H32O8	520.20972	520.2079	+H	521.2152	-3.4	175229	lignans
28	heteroclitin B		C28H34O8	498.22537	498.2254	+H, +Na	499.2327	0.1	626804	lignans
29	Kadsurindutin E	7.16	C20H24O5	344.16237	344.1631	+Na	367.1523	1.9	173712	lignans
30	schisantherin M		C32H36O10	580.23085	580.2309	+H	581.2382	0.1	295645	lignans
31	schisantherin N		C32H36O10	580.23085	580.2309	+H	581.2382	0.1	295645	lignans
32	heilaohulignans A	7.6	C26H32O8	472.20972	472.2097	+H	473.217	0	11471	lignans
33	Kadcoccinic acid A		C30H44O6	500.31379	500.3131	+H	501.3204	-1.4	265036	triterpenoids
34	Kadcoccinic acid B	7.61	C30H44O6	500.31379	500.3131	+H	501.3204	-1.4	265036	triterpenoids
35	KadcoccitoneA		C30H44O6	500.31379	500.3131	+H	501.3204	-1.4	265036	triterpenoids
36	binankadsurin A	7.77	C22H26O7	402.16785	402.169	+Na	425.1582	2.7	394112	lignans
37	kadsuphilin B		C22H26O7	402.16785	402.169	+Na	425.1582	2.7	394112	lignans
38	Longipedlactone A	7.9	C30H38O5	478.27192	478.2715	+H	479.2787	-1	132982	triterpenoids
39	KadcoccitoneC	7.98	C30H44O5	484.31887	484.3175	+H, +Na	485.3248	-2.8	401850	triterpenoids
40	KadcotrioneC		C30H44O5	484.31887	484.3175	+H, +Na	485.3248	-2.8	401850	triterpenoids
41	isokadsuranin	8.11	C23H28O6	400.18859	400.1865	+H, +Na	401.1937	-5.3	147564	lignans

Number	Component name	Observed RT (min)	Formula	Neutral mass (Da)	Observed neutral mass (Da)	Adducts	Observed m/z	Mass error (ppm)	Response	Item type
42	kadsulignans I	8.19	C25H28O8	456.17842	456.177	+H	457.1843	-3	312473	lignans
43	propoxyl oxokadsurane		C25H28O8	456.17842	456.177	+H	457.1843	-3	312473	lignans
44	isokadsuranin	8.41	C23H28O6	400.18859	400.1865	+H, +Na	401.1938	-5.2	491810	lignans
45	kadsuranin		C23H28O6	400.18859	400.1865	+H, +Na	401.1938	-5.2	491810	lignans
46	Kadcoccilactone Q	8.6	C30H42O5	482.30322	482.3021	+H, +Na	483.3093	-2.4	167720	triterpenoids
47	Kadcoccinic acid F		C30H42O5	482.30322	482.3021	+H, +Na	483.3093	-2.4	167720	triterpenoids
48	heilaohulignans C	8.71	C27H32O8	484.20972	484.2097	+Na	507.1989	0	148941	lignans
49	kadsuralignan I		C27H32O8	484.20972	484.2097	+Na	507.1989	0	148941	lignans
50	heilaohulignans C		C27H32O8	484.20972	484.2099	+Na	507.1991	0.3	203007	lignans
51	kadsuralignan I	9.07	C27H32O8	484.20972	484.2099	+Na	507.1991	0.3	203007	lignans
52	schiarisanrin A		C27H32O8	484.20972	484.2099	+Na	507.1991	0.3	203007	lignans
53	R-wuweizisu C	9.08	C22H24O6	384.15729	384.1559	+H, +Na	385.1632	-3.5	3352191	lignans
54	kadusurain C	9.45	C27H30O8	482.19407	482.1942	+H	483.2014	0.2	488848	lignans
55	14-O-demethyl polysp-erlignan D	9.73	C32H38O10	582.2465	582.2481	+Na	605.2373	2.7	175337	lignans

Number	Component name	Observed RT (min)	Formula	Neutral mass (Da)	Observed neutral mass (Da)	Adducts	Observed m/z	Mass error (ppm)	Response	Item type
56	heteroclitin D		C27H30O8	482.19407	482.1942	+H	483.2015	0.3	1236175	lignans
57	kadusurain C		C27H30O8	482.19407	482.1942	+H	483.2015	0.3	1236175	lignans
58	schiarisanrin B		C27H30O8	482.19407	482.1942	+H	483.2015	0.3	1236175	lignans
59	kadsuralignans K	9.81	C29H30O8	506.19407	506.1916	+H, +Na	507.1989	-4.8	130696	lignans
60	heteroclitin B	10.13	C28H34O8	498.22537	498.2252	+H, +Na	499.2325	-0.4	226975	lignans
61	Kadsurindutin E	10.14	C20H24O5	344.16237	344.1627	+Na	367.1519	0.8	57964	lignans
62	kadsuracoccinic acid A	10.17	C30H44O4	468.32396	468.3227	+H, +Na	469.3300	-2.7	301734	triterpenoids
63	Seconeokadsuranic acid A		C30H44O4	468.32396	468.3227	+H, +Na	469.3300	-2.7	301734	triterpenoids
64	Kadcoccilactone Q	10.48	C30H42O5	482.30322	482.3025	+H	483.3098	-1.5	233447	triterpenoids
65	Kadcoccinic acid F		C30H42O5	482.30322	482.3025	+H	483.3098	-1.5	233447	triterpenoids
66	binankadsurin A	10.76	C22H26O7	402.16785	402.1689	+Na	425.1582	2.6	289376	lignans
67	kadsuphilol A		C22H26O7	402.16785	402.1689	+Na	425.1582	2.6	289376	lignans
68	kadsuralignan A		C22H26O7	402.16785	402.1689	+Na	425.1582	2.6	289376	lignans
69	Kadcoccine acid I	11.15	C30H44O4	468.32396	468.3232	+H, +Na	469.3304	-1.7	1664514	triterpenoids
70	Kadcoccine acid K		C30H44O4	468.32396	468.3232	+H, +Na	469.3304	-1.7	1664514	triterpenoids

Number	Component name	Observed RT (min)	Formula	Neutral mass (Da)	Observed neutral mass (Da)	Adducts	Observed m/z	Mass error (ppm)	Response	Item type
71	Kadcoccinic acid C	11.78	C30H44O4	468.32396	468.3232	+H, +Na	469.3304	-1.7	1664514	triterpenoids
72	KadcoccitoneC		C30H44O5	484.31887	484.3181	+H, +Na	485.3254	-1.6	394192	triterpenoids
73	KadcotrioneC		C30H44O5	484.31887	484.3181	+H, +Na	485.3254	-1.6	394192	triterpenoids
74	Kadcoccinic acid D	12.08	C30H44O3	452.32905	452.328	+H	453.3352	-2.4	721463	triterpenoids
75	Kadcoccilactone R	12.35	C30H46O5	486.33452	486.3341	+H, +Na	487.3414	-0.8	632434	triterpenoids
76	Kadcoccinic acid G		C30H46O5	486.33452	486.3341	+H, +Na	487.3414	-0.8	632434	triterpenoids
77	kadcoccitane A		C30H46O5	486.33452	486.3341	+H, +Na	487.3414	-0.8	632434	triterpenoids
78	Kadcoccine acid I		C30H44O4	468.32396	468.3233	+H, +Na	469.3306	-1.4	2104084	triterpenoids
79	Kadcoccine acid K		C30H44O4	468.32396	468.3233	+H, +Na	469.3306	-1.4	2104084	triterpenoids
80	Kadcoccinic acid C		C30H44O4	468.32396	468.3233	+H, +Na	469.3306	-1.4	2104084	triterpenoids
81	seco-coccinic acid B	12.58	C30H46O3	454.3447	454.3427	+H	455.35	-4.3	167360	triterpenoids
82	Kadcoccilactone O	13.3	C30H36O7	508.2461	508.2487	+H	509.2559	5	52209	triterpenoids
83	heteroclic acid		C32H48O5	512.35017	512.3494	+Na	535.3386	-1.5	119198	triterpenoids
84	Kadcoccine acid A		C32H48O5	512.35017	512.3494	+Na	535.3386	-1.5	119198	triterpenoids
85	Kadcoccine acid J	13.3	C32H48O5	512.35017	512.3494	+Na	535.3386	-1.5	119198	triterpenoids

Number	Component name	Observed RT (min)	Formula	Neutral mass (Da)	Observed neutral mass (Da)	Adducts	Observed m/z	Mass error (ppm)	Response	Item type
86	Kadcoccine acid N		$C_{32}H_{48}O_5$	512.35017	512.3494	+Na	535.3386	-1.5	119198	triterpenoids
87	Kadcoccinone F		$C_{32}H_{48}O_5$	512.35017	512.3494	+Na	535.3386	-1.5	119198	triterpenoids
88	KadcoccinoneA		$C_{32}H_{48}O_5$	512.35017	512.3494	+Na	535.3386	-1.5	119198	triterpenoids
89	Kadcoccine acid F		$C_{30}H_{44}O_3$	452.32905	452.3277	+H, +Na	453.3350	-2.9	4642827	triterpenoids
90	Kadcoccine acid G		$C_{30}H_{44}O_3$	452.32905	452.3277	+H, +Na	453.3350	-2.9	4642827	triterpenoids
91	Kadcoccine acid H		$C_{30}H_{44}O_3$	452.32905	452.3277	+H, +Na	453.3350	-2.9	4642827	triterpenoids
92	Kadcoccinic acid D		$C_{30}H_{44}O_3$	452.32905	452.3277	+H, +Na	453.3350	-2.9	4642827	triterpenoids
93	Kadcoccinic acid A		$C_{30}H_{44}O_6$	500.31379	500.3135	+H	501.3208	-0.6	99780	triterpenoids
94	Kadcoccinic acid B	13.53	$C_{30}H_{44}O_6$	500.31379	500.3135	+H	501.3208	-0.6	99780	triterpenoids
95	KadcoccitoneB		$C_{30}H_{44}O_6$	500.31379	500.3135	+H	501.3208	-0.6	99780	triterpenoids
96	kadcotrione A		$C_{30}H_{44}O_6$	500.31379	500.3135	+H	501.3208	-0.6	99780	triterpenoids
97	Kadsudilactone	13.55	$C_{30}H_{44}O_4$	468.32396	468.3225	+H	469.3297	-3.2	257480	triterpenoids
98	kadcoccinic acid I	13.85	$C_{31}H_{46}O_5$	498.33452	498.3342	+H, +Na	499.3415	-0.6	1007840	triterpenoids
99	Kadcoccine acid F	13.99	$C_{30}H_{44}O_3$	452.32905	452.3282	+H, +Na	453.3355	-1.8	3415286	triterpenoids
100	Kadcoccine acid G		$C_{30}H_{44}O_3$	452.32905	452.3282	+H, +Na	453.3355	-1.8	3415286	triterpenoids
101	Kadcoccine acid H	13.99	$C_{30}H_{44}O_3$	452.32905	452.3282	+H, +Na	453.3355	-1.8	3415286	triterpenoids

Number	Component name	Observed RT (min)	Formula	Neutral mass (Da)	Observed neutral mass (Da)	Adducts	Observed m/z	Mass error (ppm)	Response	Item type
102	Kadcoccinic acid D		C30H44O3	452.32905	452.3282	+H, +Na	453.3355	-1.8	3415286	triterpenoids
103	Kadsudilactone	14.04	C30H44O4	468.32396	468.3233	+H, +Na	469.3305	-1.5	302328	triterpenoids
104	Kadsuracoccinic acid C	14.51	C30H46O4	470.33961	470.338	+H	471.3453	-3.4	100015	triterpenoids
105	kadsuric acid		C30H46O4	470.33961	470.338	+H	471.3453	-3.4	100015	triterpenoids
106	Kadsudilactone	14.65	C30H44O4	468.32396	468.3228	+H	469.3301	-2.4	342272	triterpenoids
107	Seconeokadsuranic acid A		C30H44O4	468.32396	468.3228	+H	469.3301	-2.4	342272	triterpenoids
108	seco-coccinic acid F	15.06	C30H46O4	470.33961	470.3378	+H, +Na	471.3451	-3.9	95561	triterpenoids
109	Coccinetane B		C30H48O5	488.35017	488.352	+Na	511.3412	3.5	106947	triterpenoids
110	Coccinetane C	15.86	C30H48O5	488.35017	488.352	+Na	511.3412	3.5	106947	triterpenoids
111	Kadcoccine acid L		C32H46O5	510.33452	510.3339	+H, +Na	511.3412	-1.2	106947	triterpenoids
112	Kadcoccine acid I		C30H44O4	468.32396	468.3231	+H, +Na	469.3304	-1.7	6994795	triterpenoids
113	Kadcoccine acid K	16.05	C30H44O4	468.32396	468.3231	+H, +Na	469.3304	-1.7	6994795	triterpenoids
114	Kadcoccinic acid C		C30H44O4	468.32396	468.3231	+H, +Na	469.3304	-1.7	6994795	triterpenoids
115	seco-coccinic acid J	16.19	C29H48O4	460.35526	460.3569	+Na	483.3461	3.4	3100602	triterpenoids
116	Kadcoccinic acid H	16.24	C31H48O5	500.35017	500.35	+Na	523.3392	-0.4	344343	triterpenoids

Number	Component name	Observed RT (min)	Formula	Neutral mass (Da)	Observed neutral mass (Da)	Adducts	Observed m/z	Mass error (ppm)	Response	Item type
117	kadcoccinic acid J	16.39	C31H48O5	500.35017	500.35	+Na	523.3392	-0.4	344343	triterpenoids
118	(24S)-Coccinetane C		C30H48O5	488.35017	488.3525	+Na	511.3417	4.6	417229	triterpenoids
119	Coccinetane B		C30H48O5	488.35017	488.3525	+Na	511.3417	4.6	417229	triterpenoids
120	Coccinetane C		C30H48O5	488.35017	488.3525	+Na	511.3417	4.6	417229	triterpenoids
121	Kadcoccine acid B		C32H46O5	510.33452	510.3345	+H, +Na	511.3417	-0.1	417229	triterpenoids
122	Kadcoccine acid E		C32H46O5	510.33452	510.3345	+H, +Na	511.3417	-0.1	417229	triterpenoids
123	Kadcoccine acid L		C32H46O5	510.33452	510.3345	+H, +Na	511.3417	-0.1	417229	triterpenoids
124	3-hydroxy-neokadsuranic acid	16.64	C30H46O3	454.3447	454.3436	+H	455.3509	-2.4	1003900	triterpenoids
125	coccinic acid		C30H46O3	454.3447	454.3436	+H	455.3509	-2.4	1003900	triterpenoids
126	kadcoccine acid C		C30H46O3	454.3447	454.3436	+H	455.3509	-2.4	1003900	triterpenoids
127	schisandronic acid		C30H46O3	454.3447	454.3436	+H	455.3509	-2.4	1003900	triterpenoids
128	coccinilactone B	16.91	C30H46O3	454.3447	454.3438	+H	455.351	-2.1	442707	triterpenoids
129	kadcoccine acid D		C30H46O3	454.3447	454.3438	+H	455.351	-2.1	442707	triterpenoids
130	Kadcoccine acid F	17.35	C30H44O3	452.32905	452.3278	+H, +Na	453.3351	-2.7	6549606	triterpenoids
131	Kadcoccine acid G		C30H44O3	452.32905	452.3278	+H, +Na	453.3351	-2.7	6549606	triterpenoids
132	Kadcoccine acid H	17.35	C30H44O3	452.32905	452.3278	+H, +Na	453.3351	-2.7	6549606	triterpenoids

Number	Component name	Observed RT (min)	Formula	Neutral mass (Da)	Observed neutral mass (Da)	Adducts	Observed m/z	Mass error (ppm)	Response	Item type
133	Kadcoccinic acid D		C30H44O3	452.32905	452.3278	+H, +Na	453.3351	-2.7	6549606	triterpenoids
134	Seconeokadsuranic acid A	20.81	C30H44O4	468.32396	468.3228	+H, +Na	469.3301	-2.5	185476	triterpenoids
