# Supplementary material for: UPLC-Q-TOF-MS/MS and Network Pharmacology Approaches to Explore the Active Compounds and Mechanisms of Kadsura coccinea for Treating Rheumatoid Arthritis
Source: Int J Mol Sci. 2026 Feb 24;27(5):2097. doi: 10.3390/ijms27052097 (PMC12984762; doi:10.3390/ijms27052097)
Supplement: Supplementary file 1 [file ijms-27-02097-s001.zip › 04_Table S2.pdf]

Table S2. The components related to RA targets in KC

NO.	Compound	Compound CID	Molecular Formula	Chemical Compound Classification
1	kadsuranin	3001659	C23H28O6	lignans
2	kadsuphilol A	14827760	C22H26O7	lignans
3	binankadsurin A	16757190	C22H26O7	lignans
4	kadsuralignan A	145709380	C22H26O7	lignans
5	14-O-demethyl polysp-erlignan D		C32H38O10	lignans
6	kadsuralignan I	44445502	C27H32O8	lignans
7	(5R,6R,7R)-1,2,3,10,11,12hexamethoxy-6,7-dimethyl-5,6,7,8-tetrahydrodibenzo[a,c][8]annulen-5-ol		C22H24O7	lignans
8	schisantherin M	145709273	C32H36O10	lignans
9	heteroclitin B	102004605	C28H34O8	lignans
10	diankadsurinone		C23H26O7	lignans
11	benzoylisogomisin O	91864464	C30H32O8	lignans
12	kadsuralignan K	24762751	C29H30O8	lignans
13	kadsurin	171064	C25H30O8	lignans
14	R-(+)-wuweizisu C	119112	C22H24O6	lignans
15	schisantherin N		C32H36O10	lignans
16	heilaohulignans A		C26H32O8	lignans
17	kadsuphilin B	44583794	C22H26O7	lignans
18	schiarisanrin A	145709656	C27H32O8	lignans
19	schizanrin D			lignans
20	schiarisanrin B	145709498	C24H26O8	lignans
21	isokadsuranin	158103	C23H28O6	lignans
22	acetylepigomisin R	145709320	C24H26O8	lignans
23	gomisin R	11516888	C22H24O7	lignans
24	heteroclitin D	10367978	C27H30O8	lignans
25	kadsulignan A	14352824	C23H28O7	lignans
26	coccilignan A	102142181	C22H28O7	lignans
27	heilaohulignans C			lignans
28	kadsulignan I	145709264	C25H28O8	lignans
29	kadusurain C		C27H30O8	lignans
30	isovaleroyl			lignans

	oxokadsurane			
31	benzoyl oxokadsurane	49770141	$C_{29}H_{28}O_8$	lignans
32	propoxyl oxokadsurane		$C_{25}H_{28}O_8$	lignans
33	longipedunin B	11698256	$C_{25}H_{30}O_8$	lignans
34	isovaleroyl oxokadsuranol	145709266	$C_{27}H_{32}O_9$	lignans
35	acetoxyl oxokadsurane	49770118	$C_{24}H_{26}O_8$	lignans
36	Kadsurindutin E	102142180	$C_{20}H_{24}O_5$	lignans
37	Kadcoccinic acid D	122181859	$C_{30}H_{44}O_3$	triterpenoids
38	Kadcoccinone F	145709417	$C_{32}H_{48}O_5$	triterpenoids
39	Kadcoccitone A	71517268	$C_{30}H_{44}O_6$	triterpenoids
40	Kadcoccine acid H		$C_{30}H_{44}O_3$	triterpenoids
41	Kadcoccinic acid F	145709591	$C_{30}H_{42}O_5$	triterpenoids
42	Kadcoccitone B	145709276	$C_{30}H_{44}O_6$	triterpenoids
43	Longipedlactone B	11525863	$C_{30}H_{40}O_5$	triterpenoids
44	Kadcoccine acid I	145709282	$C_{31}H_{46}O_5$	triterpenoids
45	Kadcoccine acid J	137652123	$C_{32}H_{48}O_5$	triterpenoids
46	kadcoccitane A		$C_{30}H_{46}O_5$	triterpenoids
47	kadsuracoccinic acid A	24850148	$C_{30}H_{44}O_4$	triterpenoids
48	Longipedlactone C	11656207	$C_{30}H_{40}O_6$	triterpenoids
49	Kadcoccinic acid C	122181858	$C_{30}H_{44}O_4$	triterpenoids
50	Kadcoccilactone O	145709310	$C_{30}H_{36}O_7$	triterpenoids
51	Kadcoccine acid F	137634305	$C_{30}H_{44}O_3$	triterpenoids
52	Longipedlactone E	102468392	$C_{30}H_{38}O_6$	triterpenoids
53	Kadcoccine acid A	137632967	$C_{32}H_{48}O_5$	triterpenoids
54	Kadcoccilactone Q	70697797	$C_{30}H_{42}O_5$	triterpenoids
55	Kadcoccine acid K	137640178	$C_{30}H_{44}O_4$	triterpenoids
56	kadlongilactone D	24178992	$C_{30}H_{38}O_6$	triterpenoids
57	Coccinetane B	100923237	$C_{30}H_{48}O_5$	triterpenoids
58	kadcotrione A	102131645	$C_{30}H_{44}O_6$	triterpenoids
59	Kadsudilactone	145709467	$C_{30}H_{44}O_4$	triterpenoids
60	Longipedlactone A	11605382	$C_{30}H_{38}O_5$	triterpenoids
61	kadcoccine acid C	137653691	$C_{30}H_{46}O_3$	triterpenoids
62	Kadsuracoccinic acid C	12071612	$C_{30}H_{46}O_4$	triterpenoids

63	Longipedlactone F	11705931	C30H38O6	triterpenoids
64	seco-coccinic acid F	11259251	C30H48O2	triterpenoids
65	kadsuric acid	5384417	C30H46O4	triterpenoids
