# Supplementary figures and images for: UPLC-Q-TOF-MS/MS and Network Pharmacology Approaches to Explore the Active Compounds and Mechanisms of Kadsura coccinea for Treating Rheumatoid Arthritis
Source: Int J Mol Sci. 2026 Feb 24;27(5):2097. doi: 10.3390/ijms27052097 (PMC12984762; doi:10.3390/ijms27052097)

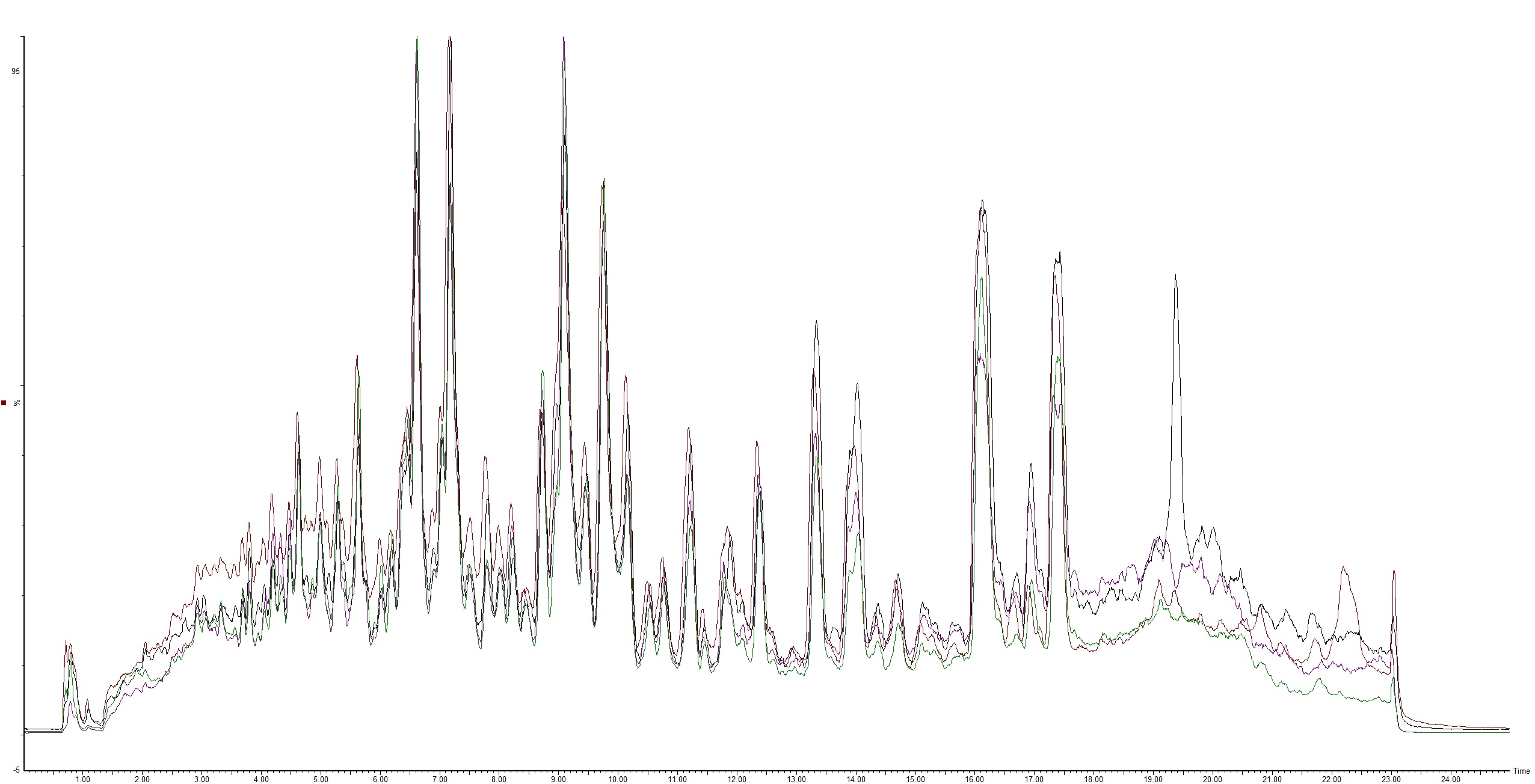

Supplement: Supplementary file 1 [file ijms-27-02097-s001.zip › Figure S1.jpg]
